# Integrated Analysis of Transcriptome and Metabolome Profiles in the Longissimus Dorsi Muscle of Buffalo and Cattle

**DOI:** 10.3390/cimb45120607

**Published:** 2023-12-04

**Authors:** Guansheng Wu, Xinjun Qiu, Zizhuo Jiao, Weijie Yang, Haoju Pan, Hong Li, Zhengyu Bian, Qiang Geng, Hui Wu, Junming Jiang, Yuanyuan Chen, Yiwen Cheng, Qiaoling Chen, Si Chen, Churiga Man, Li Du, Lianbin Li, Fengyang Wang

**Affiliations:** Hainan Key Lab of Tropical Animal Reproduction, Breeding and Epidemic Disease Research, Animal Genetic Engineering Key Lab of Haikou, School of Tropical Agriculture and Forestry, Hainan University, Haikou 570228, China; wgs2192208700@163.com (G.W.); qiuxinjun@hainanu.edu.cn (X.Q.); jiaozizhuo1998@outlook.com (Z.J.); wjyangqq@163.com (W.Y.); haoju36999@163.com (H.P.); iwbtgqm@163.com (Q.G.); 21210905000015@hainanu.edu.cn (H.W.); chen727501398@163.com (Y.C.); chensi@hainanu.edu.cn (S.C.); kych2008dl@163.com (L.D.)

**Keywords:** buffalo, meat quality, transcriptomics, metabolomics

## Abstract

Buffalo meat is gaining popularity for its nutritional properties, such as its low fat and cholesterol content. However, it is often unsatisfactory to consumers due to its dark color and low tenderness. There is currently limited research on the regulatory mechanisms of buffalo meat quality. Xinglong buffalo are raised in the tropical Hainan region and are undergoing genetic improvement from draught to meat production. For the first time, we evaluated the meat quality traits of Xinglong buffalo using the longissimus dorsi muscle and compared them to Hainan cattle. Furthermore, we utilized a multi-omics approach combining transcriptomics and metabolomics to explore the underlying molecular mechanism regulating meat quality traits. We found that the Xinglong buffalo had significantly higher meat color redness but lower amino acid content and higher shear force compared to Hainan cattle. Differentially expressed genes (DEGs) and differentially accumulated metabolites (DAMs) were identified, with them being significantly enriched in nicotinic acid and nicotinamide metabolic and glycine, serine, and threonine metabolic pathways. The correlation analysis revealed that those genes and metabolites (such as: *GAMT*, *GCSH*, *PNP*, L-aspartic acid, NADP+, and glutathione) are significantly associated with meat color, tenderness, and amino acid content, indicating their potential as candidate genes and biological indicators associated with meat quality. This study contributes to the breed genetic improvement and enhancement of buffalo meat quality.

## 1. Introduction

Meat produced from buffalo has gained increased popularity owing to its notably reduced fat and cholesterol levels, with it being considered as “the healthiest meat among red meats for human consumption” [1]. However, buffalo meat is often regarded as having inferior quality due to its darker color and reduced tenderness, with it often failing to meet the expectations of consumers [2]. Therefore, the large-scale development of the buffalo meat industry is restricted, and its enormous economic potential has not been effectively utilized. Currently, research on buffalo meat quality focuses on meat characteristics, while investigation into the underlying regulatory mechanisms of buffalo meat quality remains limited [2]. Identifying the influencing factors of key meat quality traits in buffalo is beneficial for improving the overall quality of buffalo meat.

Transcriptomics and metabolomics are effective tools in the investigation of the regulatory mechanisms underlying functional genes and differential metabolites within buffalo and cattle meat [3]. For instance, several crucial lncRNAs such as MSTRG.48330.7, MSTRG.30030.4, and MSTRG.203788.46 were identified through RNA-seq analysis, which may have an impact on the growth of buffalo muscles [4]. A study was conducted utilizing the GC-MS metabolomics technique to investigate the differential changes in metabolites of buffalo rumen under heat stress conditions [5]. At present, there is no application of metabolomics in assessing buffalo meat quality. The single omics technique of RNA-seq is not sufficient for a clear understanding of biological processes, and metabolomics can further analyze the auxiliary metabolic changes caused by post-transcriptional regulation to accurately reflect the physiological status of the organism [6,7]. Therefore, this study aimed to jointly analyze transcriptomics and metabolomics data of Xinglong buffalo and Hainan cattle to explore the underlying regulatory mechanisms in meat quality differences.

The Xinglong buffalo is an indigenous river-type buffalo breed in Hainan, with heat and humidity resistance, disease resistance, and other characteristics, with it being a meet buffalo breed. Hainan cattle, similar to the Xinglong buffalo, inhabit the Hainan region. As the main indigenous beef cattle breed, Hainan cattle are favored by local consumers because of their good meat quality and fresh taste [8,9]. This study used Hainan cattle as a control and selected Xinglong buffalo and Hainan cattle grazing in the same area as experimental subjects. We aimed to identify the key factors that caused the differences in meat quality characteristics between Xinglong buffalo and Hainan cattle through transcriptomics and metabolomics, in order to reveal the distinction between molecular regulatory mechanisms. It will provide important insights into buffalo meat quality regulation and serve as a reference for molecular breeding in buffalo meat production.

## 2. Materials and Methods

### 2.1. Experimental Animals and Sample Collection

Eight healthy Hainan cattle and eight healthy Xinglong buffalo, with an average age of 18 months, were randomly selected and evenly divided into two groups (H group, *n* = 8, all males; X group, *n* = 8, all males). All of the animals had been castrated. Both groups were grazed freely on the same local grass in Ding’an County without supplemental feeding from the time they were weaned at 3 months old until they were slaughtered at 18 months of age. Approximately 45 min after slaughter, the longissimus dorsi (only a steak) between the twelfth and thirteenth ribs were chosen for on-site quality assessment. One-half of the muscle samples were frozen in liquid nitrogen and then stored at −80 °C for total RNA and metabolite extraction (H group: *n* = 4; X group: *n* = 4). Additionally, the rest of the muscle samples were placed in an icebox and transported back to the laboratory for storage in a −20 °C refrigerator for the determination of crude fat (Soxhlet extractor, Jinan, China), crude protein content (BUCHI automatic Kjeldahl nitrogen analyzer K-375, Shanghai, China), and amino acid composition (LC-MS), among other meat quality indicators (H group: *n* = 4; X group: *n* = 4). All procedures were approved by the Academic Committee of Hainan University, following Animal Welfare and Ethical guidelines (approval code: HNUAUCC-2022-00010).

### 2.2. Meat Quality Determination

According to the method of Hoa et al. [10], the color of the meat was measured along the cross-section of the samples using a portable colorimeter, and the average value was taken three times at different positions. The pH values of the samples were measured using a pH meter, which can be inserted into the meat immediately, at 45 min and 24 h after slaughter. Similar to the color measurement, the average value was taken three times at different positions. The drip loss was determined according to the description by Wang et al. [11]. Firstly, the muscle was trimmed along the fiber direction into strips of 5 cm × 3 cm × 2 cm in length, width, and height and weighed (W1). Then, the meat strip was hooked by a fine iron wire at one end hung vertically in a foam box and refrigerated at 4 °C for 24 h. We measured the weight (W2) and calculated the drip loss using the following formula. Drip loss (%) = [(W1 − W2)/W1] × 100%. Each sample was repeated three times, and the average result was taken. The shear force of the muscle samples was determined by referring to the method of Huang et al. [12]. The muscle samples were trimmed to 6 cm × 3 cm × 3 cm and then placed in a constant-temperature water bath. The samples were heated until the thermometer measured the center temperature of the meat block as 70 °C and continued to cook at this temperature for an additional 20 min. After cooling to room temperature, samples were taken along the direction of muscle fiber growth using a circular punch sampler with a diameter of 1.27 cm, and the shear force was then measured using a shear instrument. The measurement was repeated five times, and the average value was taken. Crude protein content was determined by following the methods by Bai et al. [13], and crude fat, crude ash, and moisture content were determined by referring the methods of Bostami et al. [14]. The amino acid content of the muscle samples was measured following the method of Wang et al. [15].

### 2.3. RNA-Seq and Transcriptome Data Analysis

Total RNA was extracted from the muscle samples following the instructions for TRlzol Reagent (Life Technologies, Carlsbad, CA, USA). The quality of RNA was assessed using a NanoDrop 2000 (Thermo Fisher Scientific, Wilmington, DE, USA), while the integrity of the RNA was evaluated using an Agilent Bioanalyzer 2100 system (Agilent Technologies, Santa Clara, CA, USA) with a RNA Nano 6000 detection kit. Biomarker Technologies Co., Ltd. (Beijing, China) carried out the construction of gene libraries and RNA-seq using qualified RNA samples. After the libraries passed quality control, we proceeded with PE150 pattern sequencing by utilizing the Illumina NovaSeq6000 sequencing platform. To obtain clean data, we employed perl scripting to remove reads that contained connectors and low-quality reads (reads with a proportion of N > 10% or a proportion of bases with Q ≤ 10 exceeding 50%) from the raw data. The clean data were compared with the reference genome (Bos_taurus_UMD_3.1.1) using Hisat2 version 2.0.4. The overview of the sequencing reads alignment to the reference genome was presented in Table S1. Gene expression levels were quantified by fragments per kilobase per million reads (FPKM), and differential gene expression analysis of the two groups of samples was conducted through the utilization of DESeq2 version 1.30.1. Genes with a |log2 (fold change, FC)| > 1 and a false discovery rate (FDR) < 0.01 were deemed DEGs. The function of DEGs was annotated through databases such as Gene Ontology (GO) and the KEGG Ortholog database (KO). The DEGs underwent GO enrichment analysis and Kyoto Encyclopedia of Genes and Genomes (KEGG) pathway enrichment analysis using clusterProfiler version 4.4.4.

### 2.4. Quantitative Real-Time Polymerase Chain Reaction (qRT-PCR)

The extracted total RNA from the samples was converted to cDNA through reverse transcription, and the validation of the selected DEGs was conducted using qRT-PCR. The *GAPDH* gene was used as the internal reference gene. The Primer-BLAST tool of the NCBI was utilized to design specific primers for the DEGs, and the relevant gene names and primer sequences were in Table S2. The qRT-PCR was carried out using the Biosharp real-time fluorescence quantitative reagent kit on the QuantStudio^TM^ 5 Real-Time PCR Instrument (Thermo Fisher Scientific, Wilmington, DE, USA). The program used was as follows: 5 min at 95 °C; 10 s at 95 °C; and 30 s at 60 °C for 40 cycles. The relative expression levels of the genes were determined using the 2^−ΔΔCt^ method [16], and statistical analysis was conducted using SPSS version 20.0 with the one-way analysis of variance (ANOVA). Significance was defined as *p*-value < 0.05.

### 2.5. LC-MS/MS Metabolomics Analysis

Initially, a muscle sample weighing 50 mg and 1000 μL of an extract containing an internal standard (L-2-chlorophenylalanine at a concentration of 20 mg/L) was introduced and agitated for 30 s. After complete blending, steel beads were introduced and ground at a frequency of 45 Hz for a duration of 10 min, followed by sonication in an ice-water bath for another 10 min. The samples were placed at −20 °C for 1 h and then subjected to centrifugation at 4 °C for 15 min at 12,000 rpm. Next, 500 μL of supernatant was taken out in a tube and dried, followed by the addition of 160 μL of extraction solution was added to redissolve the sample. The samples were vortexed for 30 s, the centrifugation and ultrasonic treatment process described above was repeated, and the supernatant was then collected for LC-MS/MS analysis.

The liquid chromatography system and chromatographic column employed for the study were identical to those utilized by Wang et al. [17]. The liquid chromatographic mobile phase conditions were as follows: positive ion mode (POS) and negative ion mode (NEG) mobile phase A was 0.1% formic acid aqueous solution, and mobile phase B was a 0.1% formic acid acetonitrile solution; at 0–10 min, 98% A phase and 2% B phase; at 10–13 min, 2% A phase and 98% B phase; and at 13–15 min, 98% A phase and 2% B phase. The flow rate was 400 μL/min, and the injection volume was 1 μL. The ESI ion source operating parameters were as follows: capillary voltage of 2500 V (POS) or −2000 V (NEG); cone hole voltage of 30 V; ion source temperature of 100 °C; desolvent gas temperature of 500 °C; backblast gas flow rate of 50 L/h; desolvent gas flow rate: 800 L/h; and mass to nucleus ratio (*m*/*z*) acquisition range of 50–1200. Finally, the acquisition of data was carried out using the MSe mode controlled by MassLynx version 4.2. During each cycle, simultaneous dual-channel data acquisition was carried out for both low and high collision energies. The lower one was set at 2 V, while the higher one spanned from 10 to 40 V. The scan frequency utilized for generating one mass spectral map was 0.2 s.

The raw data were subjected to processing through the utilization of Progenesis QI version 3.0, such as peak extraction, alignment, and other related procedures. The processed data were then compared and annotated with relevant databases (including KEGG, HMDB, and LIPID MAPS databases). Metabolite accumulation specificity was studied using principal component analysis (PCA) and orthogonal partial least-squares discriminant analysis (OPLS-DA). Metabolites with significant differences were screened using a threshold of variable important in projection (VIP) ≥ 1, *p*-value < 0.05, and |log2 FC| > 1, and the significantly changed differentially accumulated metabolites (DAMs) were then analyzed for metabolic pathway and functional enrichment using the KEGG database.

### 2.6. Transcriptome and Metabolome Joint Analysis

To integrate the transcriptomic and metabolomic data, R was used to calculate Pearson correlation coefficients between the DEGs and DAMs [18]. DEGs and DAMs with |r| >0.80 and a *p*-value < 0.05 were filtered out based on strong correlations, and the KEGG database was used for joint biological annotation. Cytoscape version 3.9.1 was used to visualize the network relationships between differentially expressed genes and metabolites in the significantly enriched pathways [19]. Furthermore, the correlation between some DEGs and DAMs and phenotypic traits was researched through Pearson correlation analysis.

### 2.7. Statistical Analysis

All data were represented as the mean ± standard deviation (SD). Statistical analysis was conducted using SPSS version 20.0 with the one-way analysis of variance (ANOVA). Significance was defined as a *p*-value < 0.05. GraphPad Prism version 8.0.2 was used to visualize the qRT-PCR results. Cytoscape version 3.9.1 was used to visualize the network relationships between DEGs and DAMs.

## 3. Results

### 3.1. Analysis of Meat Characteristics and Amino Acid Composition

The Xinglong buffalo group showed significantly higher a* values (14.14 ± 1.05 vs. 12.77 ± 1.12, *p* < 0.05) and shear force (76.77 ± 7.47 N vs. 58.14 ± 16.14 N, *p* < 0.05) compared to the Hainan cattle group, while the other meat quality traits showed no notable disparities between the two groups. The X group also exhibited significantly lower levels of total amino acids (TAAs), essential amino acids (EAAs), non-essential amino acids (NEAAs), and flavor amino acids (FAAs) compared to the H group (*p* < 0.05) (Table 1). According to the amino acid composition analysis, it was observed that the X group had significantly lower levels of six EAAs, four FAAs, and one NEAA (Table 2).

### 3.2. Analysis of DEGs and Transcriptome Data

**Figure 1:**
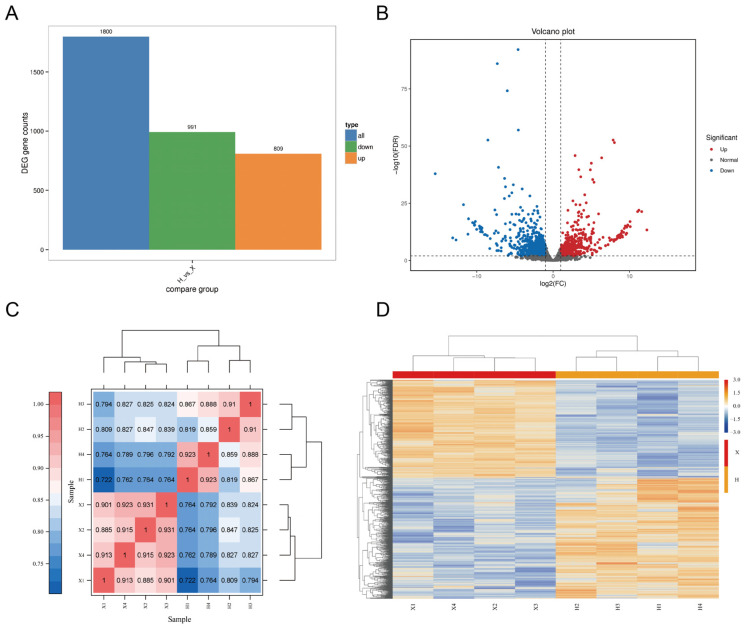
DEGs expression profiles of Xinglong buffalo and Hainan cattle. (**A**) Histogram of the number of DEGs. (**B**) Volcano plot of DEGs. (**C**) Heat map of sample correlation. (**D**) Heat map of DEGs clustering.

**Figure 2:**
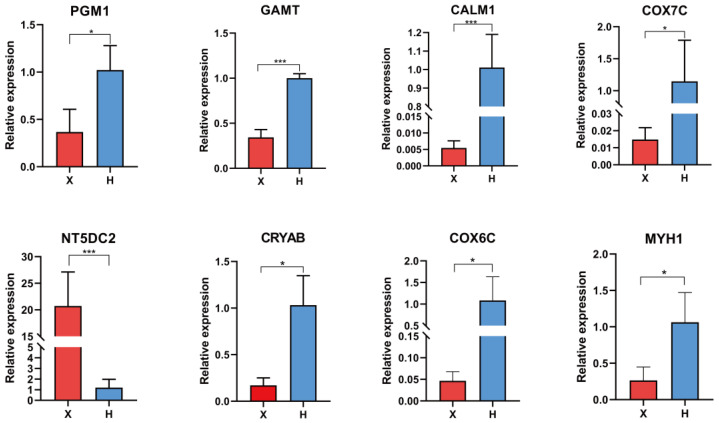
qRT-PCR analysis of the selected DEGs in Xinglong buffalo and Hainan cattle. (* indicates *p* < 0.05, *** indicates *p* < 0.001).

All samples had a Q30 above 94.83% and 6.11 Gb of clean data. The mapping efficiency was 69.54–74.29% for group X and 94.96–95.90% for group H (Table S1). A total of 1800 DEGs (809 upregulated and 991 downregulated) were identified between the groups (Figure 1A, Table S3). The distribution of DEGs is shown in Figure 1B. The heat map of sample correlation showed good repeatability within each group (Figure 1C), while the clustered expression heat map of DEGs indicated a similar expression trend within each group (Figure 1D). The qRT-PCR validated that eight randomly chosen DEGs showed consistent expression changes with the RNA-seq data (Figure 2).

**Figure 3:**
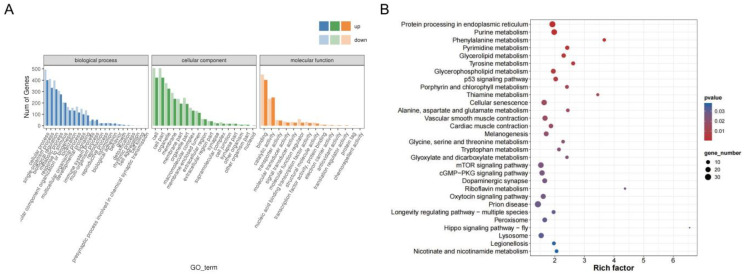
Functional annotation analysis of the DEGs. (**A**) GO functional annotations of the DEGs. (**B**) The top 30 KEGG enrichment pathways of the DEGs.

The identified DEGs were classified and functionally annotated using GO analysis, revealing predominant enrichment in biological process (BP), followed by cellular component (CC), and comparatively lower enrichment in molecular function (MF). The DEGs in the functional categories of the BP were mainly enriched in the cellular process, in the CC, this was mainly the cells, and in the MF, this was binding (Figure 3A). The KEGG pathway enrichment analysis revealed that DEGs exhibited enrichment in a total of 323 pathways, of which 34 were significantly enriched and primarily associated with amino acid metabolism and protein processing (Figure 3B). The DEGs in glycine, serine, and threonine metabolism as well as nicotinic acid and nicotinamide metabolism were significantly enriched. These pathways were closely related to meat color and tenderness, suggesting that they may be implicated in meat quality differences.

### 3.3. Analysis of DAMs and Metabolomic Data

**Figure 4:**
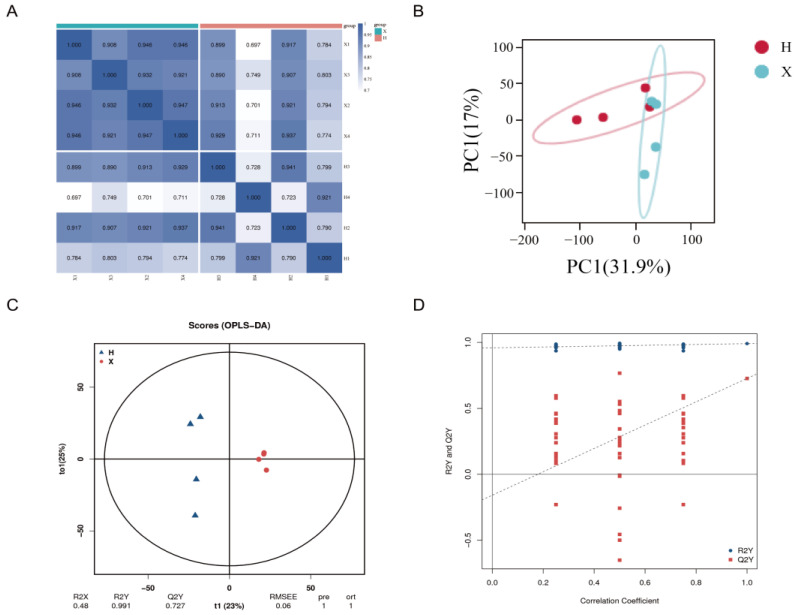
The DAMs between group H and group X. (**A**) Heat map of sample correlations. (**B**) Principal component analysis (PCA) of the samples. (**C**) OPLS-DA model score plot. (**D**) Scatter plot of OPLS-DA model.

A total of 2206 metabolites were detected in two sets of muscle samples, and 95 upregulated and 20 downregulated metabolites were identified as DAMs after screening. The heatmap of sample correlation displayed good reproducibility within each group (Figure 4A). PCA indicated that all samples were within the 95% confidence interval, and there was a certain degree of separation between groups (Figure 4B). The OPLS-DA model illustrated that there were differences between the two groups, with R2Y = 0.991 and Q2Y = 0.727, indicating that the model was valid and reliable (Figure 4C,D).

**Figure 5:**
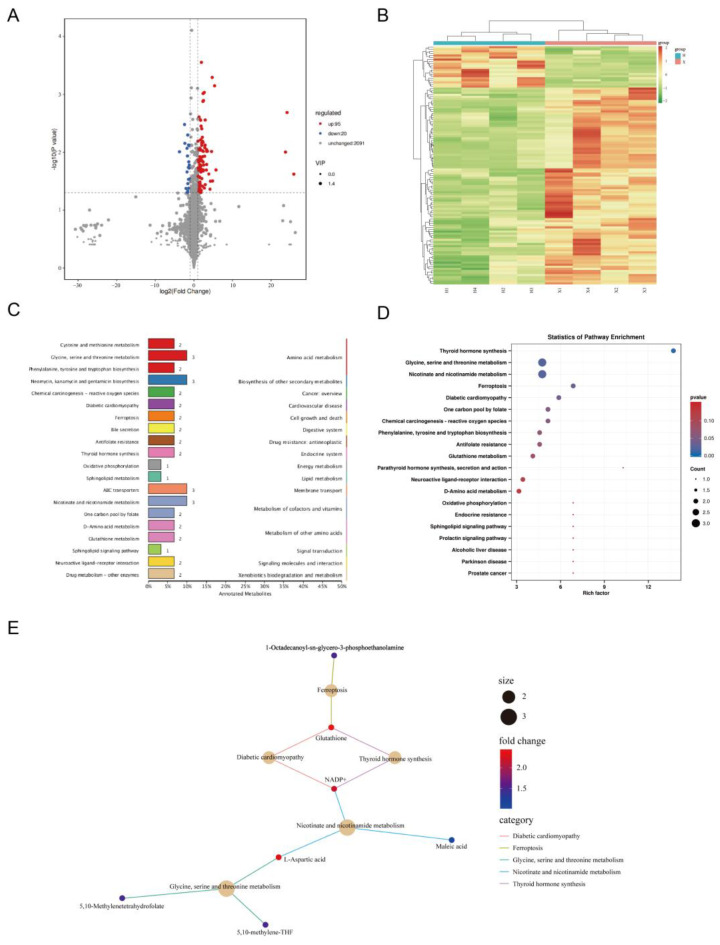
Functional annotation analysis of the DAMs. (**A**) Volcano diagram of the DAMs. (**B**) Heat map of DAMs clustering. (**C**) Top 20 entries of KEGG pathway annotations of the DAMs. (**D**) Top 20 KEGG-enriched pathways of the DAMs. (**E**) Plots of the five significantly enriched pathways and associated DAM enrichment networks (*p* < 0.05).

The total annotated metabolites and the DAMs were identified between the H and X groups were listed in Tables S4 and S5. The distribution of DAMs was depicted in Figure 5A. The clustering heatmap of DAMs indicated that the muscle samples within the group exhibited similar expression tendencies (Figure 5B). DAMs were annotated to 60 KEGG pathways, among which amino acid metabolism, vitamin and cofactor metabolism, and secondary metabolite synthesis were the main entries (Figure 5C). KEGG enrichment analysis revealed that five pathways were significantly enriched, including thyroid hormone synthesis, glycine, serine, and threonine metabolism, nicotinate and nicotinamide metabolism, ferroptosis, and diabetic cardiomyopathy (Figure 5D). The interaction between the five significantly enriched pathways and the six DAMs enriched in those pathways was illustrated in Figure 5E. In particular, some DAMs participated in multiple pathways, such as L-aspartic acid, NADP+, and glutathione (which were all higher in the X group).

### 3.4. Joint Analysis of Transcriptome and Metabolome Data

**Figure 6:**
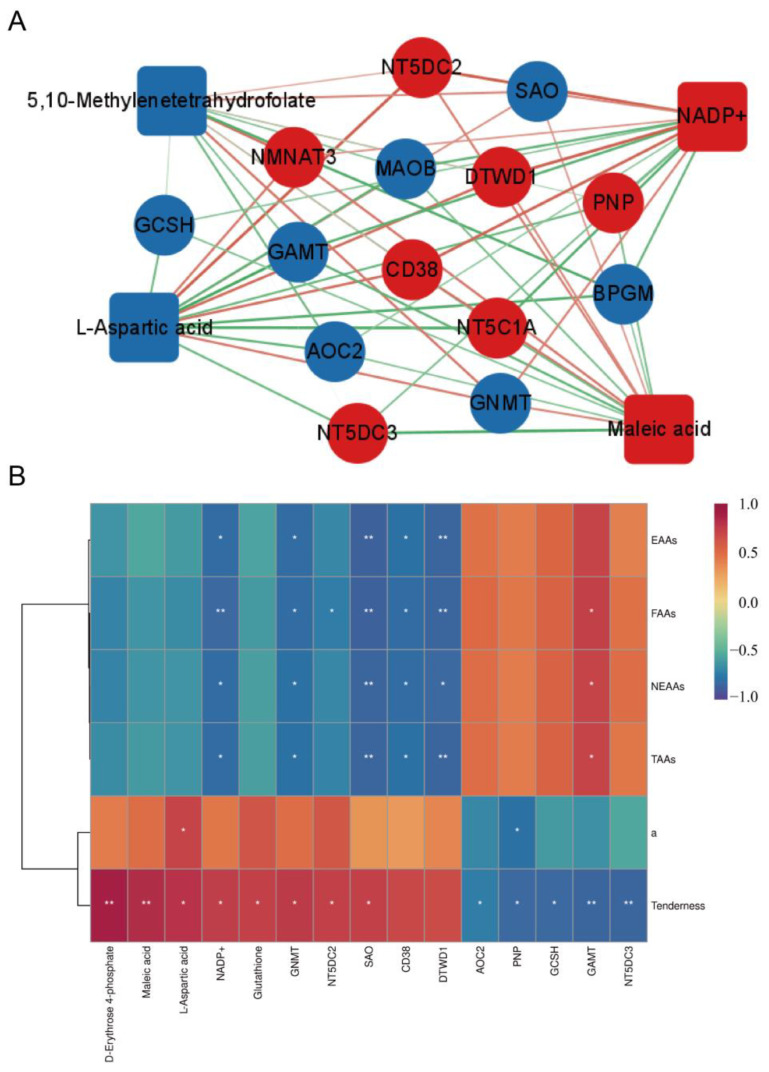
Correlation analysis. (**A**) Network diagram interactions of genes and metabolites. (**B**) Heat map of correlations of some DEGs and DAMs with phenotypic traits (* indicates *p* < 0.05; ** indicates *p* < 0.01).

The integrated analysis identified glycine, serine, and threonine metabolism and nicotinate and nicotinamide metabolism pathways enriched in both transcriptome and metabolome profiles (Table S6). A total of 16 DEGs and 4 DAMs were enriched in these two pathways, and the interaction network among these genes and metabolites was shown in Figure 6A (two DEGs were not annotated, so only the network interactions of the remaining 14 DEGs with DAMs were shown). We conducted a correlation analysis between these DEGs and DAMs (Table S7) with differential phenotypic traits. Furthermore, the DAMs in the other three significant enrichment pathways of the metabolome were also examined during correlation analysis with the differential phenotypic traits. The correlation analysis showed that some DAMs and DEGs were significantly correlated with meat quality traits (Figure 6B).

There was a significantly positive correlation (*p* < 0.05) between muscle tenderness and the metabolites D-Erythrose 4-phosphate, maleic acid, L-aspartic acid, NADP+, glutathione (which are all higher in the X group), as well as the genes *GNMT*, *NT5DC2*, and *SAO*. On the other hand, the genes *NT5DC3*, *GAMT*, *GCSH*, *PNP*, and *AOC2* exhibited a noteworthy negative correlation (*p* < 0.05) with tenderness. Moreover, L-aspartic acid demonstrated a significantly positive correlation (*p* < 0.05) with meat color redness (a* values), while *PNP* displayed a significantly negative correlation (*p* < 0.05) with it. Additionally, NADP+, *GNMT*, *SAO*, *CD38*, and *DTWD1* were all significantly negatively correlated (*p* < 0.05) with EAAs, FAAs, NEAAs, and TAAs. Conversely, *GAMT* exhibited a noteworthy positive correlation (*p* < 0.05) with EAAs, FAAs, NEAAs, and TAAs.

## 4. Discussion

Buffalo have the advantages of strong resistance to disease, adaption to different climatic conditions and management systems, and the ability to digest low-quality forage while still exhibiting a relatively faster growth rate [20]. Buffalo meat is becoming increasingly popular due to its lower fat and cholesterol content, and it is also an important source of protein for people [21]. With the advancement of technology and the popularization of agricultural mechanization, the utilitarian value of buffalo has been gradually lost [22]. Therefore, there is an urgent need for research into enhancing meat quality. Meat quality is a complex characteristic determined by many physicochemical features, such as pH, tenderness, meat color, amino acid composition, and flavor [23]. This study aimed to compare the meat quality of Xinglong buffalo and Hainan cattle under similar conditions. We observed higher a* values but lower amino acid content and higher shear force in the buffalo, suggesting lower meat quality. These results are in line with previous studies showing the poorer meat quality of buffalo [4,24]. Differences in meat color, tenderness, and amino acid composition may account for this phenomenon and could be potential targets for buffalo meat quality improvement.

The main meat characteristic that drives consumer purchasing is meat color [25]. In this study, Xinglong buffalo meat exhibited a significantly higher a* value, indicating higher redness. The transcriptomic and metabolomic data revealed that both omics KEGG pathways were significantly enriched in niacin and nicotinamide metabolism. Zhan et al. found that the downregulation of two key genes in this pathway could reduce NAD+ intermediates, thereby regulating meat color in pigs [26]. Nicotinamide can help combat free radicals and reduce their damage to cells, acting as an antioxidant enzyme and contributing to the maintenance of meat color [27]. The upregulation of *NMNAT3* and *DTWD1* in our study may increase NADP+ content, potentially influencing niacinamide content, thus regulating meat color. Additionally, the downregulation of the *PNP* gene may contribute to changes in niacin content, which could also be a factor leading to variations in meat color. Correlation analysis showed that *PNP* was significantly negatively correlated with a* value while L-aspartic acid was positively correlated, suggesting they may regulate buffalo meat color by influencing niacin and niacinamide metabolism. Furthermore, muscles with a low pH have more light scattering, which can explain the observed high lightness in this study. Meanwhile, the concentration of myoglobin and the content of deoxymyoglobin are also important factors that affect the color of meat [28,29]. In summary, genes and metabolites in the niacin and niacinamide metabolism pathway likely play important regulatory roles in meat color differences between Xinglong buffalo and Hainan cattle. Additional experimental validation for the underlying mechanisms is warranted.

Tenderness, generally measured by the magnitude of shear force, is considered another main factor that influences the purchase inclination of consumers. The tenderness of meat is influenced by numerous factors (such as species, animal age, and breed), and changes in muscle metabolism are the main factors [30,31]. In this study, Xinglong buffalo meat exhibited significantly higher shear force, indicating poorer tenderness. The transcriptomic and metabolomic data revealed that both omics KEGG pathways were significantly enriched in glycine, serine, and threonine metabolism, which may be related to tenderness. Glycine is the main structural unit of connective tissue and can bind with collagen protein to enhance the tenderness of meat [32]. Threonine is an important component of myofibrillar protein and collagen protein, which contributes to the increase in tenderness and flavor [33]. The amino acid content in group X was significantly lower, which may be the reason for the poor tenderness of buffalo meat. The correlation analysis found that D-Erythrose 4-phosphate, L-aspartic acid, glutathione, and several genes (e.g., *NT5DC3*, *GAMT*, and *GCSH*) were significantly correlated with tenderness. Zhang et al. found that D-Erythrose 4-phosphate can regulate meat quality through the glycolysis pathway [34]. Research by Chen et al. suggested that L-aspartic acid may affect meat quality by influencing the metabolism of the intestinal microbiota [35]. Additionally, it has been reported that the gene *GAMT* may improve meat quality by catalyzing creatine synthesis [36,37]. In conclusion, differences in amino acid and energy metabolism likely underlie the higher shear force of Xinglong buffalo meat. Further exploration of key genes and metabolites is needed to identify potential targets for improving tenderness.

The composition and content of amino acids are important meat characteristics [38,39]. As described above, the lower glycine and threonine content may be the reason for the poor tenderness of buffalo meat. In this study, the content of four FAAs (arginine, glycine, aspartic acid, and glutamic acid) in Xinglong buffalo was significantly lower than those in Hainan cattle, while the overall protein content was similar. These amino acids contribute to the freshness and sweetness of meat, as well as its overall acceptability, which explains why Hainan cattle are more favored by locals [40,41]. The correlation analysis found that NADP+, *GNMT*, and *SAO* were significantly correlated with FAAs. Furthermore, these genes and metabolites were significantly enriched in amino acid metabolism pathways, possibly affecting the amino acid content. Proline is a component of collagen, which helps maintain moisture in muscles, enhances flavor, and improves tenderness [42]. Lysine and methionine are both components of myofibrillar proteins. In addition, lysine helps retain water, while methionine regulates fat metabolism [43,44]. Tyrosine can produce compounds like tyrosine ketone and tyrosine alcohol through oxidation, which can impact the color and flavor of meat [45]. These amino acid contents were all lower in the X group, indicating the poor meat quality of buffalo, mainly in terms of tenderness and flavor. In summary, the difference in amino acid metabolism may be the reason for the low amino acid content of Xinglong buffalo meat. The molecular mechanism of regulating the biosynthesis of amino acids needs to be further explored.

## 5. Conclusions

Overall, we compared the differences in meat quality between Hainan cattle and Xinglong buffalo and revealed their differences in transcription and metabolic levels through the combined analysis of transcriptome and metabolome profiles. This study contributes to the genetic improvement of breeds and the enhancement of buffalo meat quality. However, further research is needed to determine the specific regulatory mechanisms of meat quality characteristics in Hainan cattle and Xinglong buffalo.

## Figures and Tables

**Table 1:** Analysis of meat quality characteristics and amino acids.

Item			H	X	*p*-Value
Meat quality characteristics	pH	45 min	6.22 ± 0.13	6.21 ± 0.18	0.918
		24 h	5.29 ± 0.31	5.14 ± 0.08	0.229
	Peat color	L	30.59 ± 1.21	30.11 ± 1.60	0.536
		a	12.77 ± 1.12 a	14.14 ± 1.05 b	0.034
		b	5.55 ± 1.13	5.23 ± 0.95	0.569
	Shear force (N)		58.14 ± 16.14 a	76.77 ± 7.47 b	0.015
	Drip loss (%)		3.77 ± 1.06	4.77 ± 1.20	0.121
	Moisture (%)		70.59 ± 2.68	73.69 ± 0.91	0.108
	Ash (%)		4.33 ± 0.03	4.52 ± 0.09	0.206
	Crude fat (%)		3.31 ± 1.44	2.90 ± 0.66	0.667
	Crude protein (%)		25.89 ± 2.32	23.00 ± 0.68	0.083
Amino acids	TAA (g/100 g)		16.09 ± 0.74 a	13.63 ± 1.39 b	0.035
	EAA (g/100 g)		6.50 ± 0.29 a	5.44 ± 0.62 b	0.036
	NEAA (g/100 g)		9.59 ± 0.46 a	8.19 ± 0.77 b	0.036
	FAA (g/100 g)		6.76 ± 0.32 a	5.71 ± 0.52 b	0.026

H: Hainan cattle; X: Xinglong buffalo. TAAs: total amino acids; EAAs: essential amino acids (Lys + Met + Thr + Val + Tyr + Phe + His + Arg); NEAAs: non-essential amino acids (TAAs − EAAs); FAAs: flavor amino acids (Glu + Asp + Ala + Arg + Gly). Different lowercase letters in the same row indicate significant differences (*p* < 0.05).

**Table 2:** Analysis of amino acid compositions.

Amino Acids (mg/100 g)	H	X	*p*-Value
Histidine	703.28 ± 26.78	618.66 ± 85.59	0.153
4-Hydroxy-L-Proline	62.96 ± 13.48	53.77 ± 4.68	0.307
Arginine	1035.82 ± 46.33 a	863.49 ± 91.08 b	0.027
Asparagine	0.18 ± 0.09	0.12 ± 0.03	0.327
Glutamine	0.97 ± 0.45	0.53 ± 0.06	0.145
Serine	652.55 ± 41.72	560.99 ± 56.45	0.065
Glycine	935.51 ± 50.60 a	813.65 ± 54.36 b	0.029
Aspartic acid	1612.44 ± 78.86 a	1346.59 ± 129.67 b	0.023
Glutamic acid	1913.71 ± 94.63 a	1600.13 ± 146.62 b	0.021
Threonine	845.14 ± 39.25 a	722.31 ± 71.07 b	0.040
Alanine	1258.15 ± 73.11	1084.39 ± 104.57	0.056
Gamma-Aminobutyric acid	1.44 ± 0.35	1.07 ± 0.15	0.148
Proline	892.72 ± 55.23 a	763.97 ± 71.79 b	0.049
D-2-Aminobutyric acid	0.24 ± 0.15	0.25 ± 0.09	0.932
Lysine	1409.18 ± 103.03 a	1114.90 ± 178.08 b	0.048
Cystine	21.16 ± 2.89	16.33 ± 2.30	0.064
Methionine	391.74 ± 24.23 a	325.51 ± 30.01 b	0.025
Tyrosine	667.48 ± 32.20 a	545.68 ± 57.27 b	0.018
Valine	711.07 ± 23.14	614.74 ± 66.46	0.055
Isoleucine	732.46 ± 17.87	639.48 ± 69.92	0.067
Leucine	1503.17 ± 61.82	1309.57 ± 139.75	0.071
Phenylalanine	737.14 ± 30.88 a	631.02 ± 68.46 b	0.050
Tryptophan	6.40 ± 5.11	0.90 ± 0.09	0.112

H: Hainan cattle; X: Xinglong buffalo. Different lowercase letters in the same row indicate significant differences (*p* < 0.05).

## Data Availability

The datasets presented in this study can be found in online repositories. The raw sequence data have been deposited in the GSA of the CNCB, accession number: CRA012686 (https://ngdc.cncb.ac.cn/gsa, accessed on 18 September 2023).

## Supplementary Materials

The following supporting information can be downloaded at: https://www.mdpi.com/article/10.3390/cimb45120607/s1.
