# Supplementary material for: Integrated Analysis of Transcriptome and Metabolome Profiles in the Longissimus Dorsi Muscle of Buffalo and Cattle
Source: Curr Issues Mol Biol. 2023 Dec 4;45(12):9723–36. doi: 10.3390/cimb45120607 (PMC10741837; doi:10.3390/cimb45120607)
Supplement: Supplementary file 1 [file cimb-45-00607-s001.zip › Table S1-S2.pdf]

**The Supplementary Table S1-S2**

Table S1.Overview of the sequencing reads alignment to the reference genome

| Sample | Clean Reads | Mapped Reads | Uniq Mapped Reads | Multiple Map Reads | GC Content | (%)≥Q30 |
|---|---|---|---|---|---|---|
| H1 | 48,911,196 | 46,744,271 (95.57%) | 45,332,170 （92.68%） | 1,412,101 (2.89%) | 53.84% | 95.46% |
| H2 | 52,626,036 | 49,974,510 (94.96%) | 48,425,710 （92.02%） | 1,548,800 (2.94%) | 53.20% | 95.52% |
| H3 | 48,352,530 | 46,370,897 (95.90%) | 45,006,450 （93.08%） | 1,364,447 (2.82%) | 53.42% | 95.39% |
| H4 | 46,259,594 | 44,115,880 (95.37%) | 42,752,634 （92.42%） | 1,363,246 (2.95%) | 53.11% | 95.25% |
| X1 | 46,758,086 | 32,513,488 (69.54%) | 31,664,157 （67.72%） | 849,331 (1.82%) | 50.80% | 95.01% |
| X2 | 40,761,760 | 30,283,325 (74.29%) | 29,279,572 （71.83%） | 1,003,753 (2.46%) | 52.26% | 94.82% |
| X3 | 50,006,008 | 36,339,224 (72.67%) | 35,190,724 （70.37%） | 1,148,500 (2.30%) | 52.35% | 95.17% |
| X4 | 53,124,936 | 38,975,952 (73.37%) | 37,826,214 （71.20%） | 1,149,738 (2.16%) | 52.35% | 95.22% |

| Table S2. Primer information | | | |
|---|---|---|---|
| Gene | Accession No. | Primer sequences (5'-3') | Products length (bp) |
| CRYAB | NM_174290.2 | F:ATGAAGAGCGCCAGGATGAAC<br>R:AGTAATGGCGAGAGGGTCCA | 92 |
| GAMT | NM_001038544.2 | F:ACCCTGCCAGACAGTCACTT<br>R:GGAAGGCATGGTCCCGAATG | 106 |
| NT5DC2 | XM_003587635.2 | F:ACTTGATGAGAAGGGCTCGC<br>R:GTCAAACAGGTTTCCCTGCC | 85 |
| CALM1 | NM_001242572.1 | F:GGCACCATTGACTTCCCAGA<br>R:ACCCGTTGCCATCCTTATCG | 115 |
| MYH1 | XM_010815980.3 | F:TACCCTGGGGATGAGTATGAC<br>R:ATCAAAGACCCTGCCTTGGAG | 91 |
| COX6C | NM_001244111.1 | F:TTTGGACTCTTTCGGCCACC<br>R:TTCTTCTCGACGACTCAACCG | 131 |
| COX7C | XM_005209728.4 | F:GTGAGGTAAGGAGCGGTAG<br>R:ATAGTGGCTCCGACGAAC | 140 |
| PGM1 | NM_001076903.1 | F:GGGGACGGCAGGTTCTACAT<br>R:AACCAAGCGACCAATCCCG | 81 |
